# Many faces of Wilms Tumor: Recent advances and future directions

**DOI:** 10.1016/j.amsu.2021.102202

**Published:** 2021-03-07

**Authors:** Namita Bhutani, Pradeep Kajal, Urvashi Sharma

**Affiliations:** a Deptt. of Pathology, North DMC Medical College and Hindu Rao Hospital, New Delhi, India; b Deptt. of Paediatric Surgery, PGIMS Rohtak, Haryana, India

**Keywords:** Kidney neoplasms, Nephroblastoma, NWTS, Pediatric malignancy, Wilms' tumor

## Abstract

**Background:**

Wilms’ tumor (WT) is the most frequently occurring paediatric renal tumor and is one of the most treatment-responsive tumors. A tumor-suppressor gene and other genetic abnormalities have been implicated in its etiology. In addition, patients with many congenital anomalies, such as Beckwith-Wiedemann syndrome, WAGR syndrome and Denys-Drash syndrome, have an increased risk of WT.

**Methods and results:**

Two large collaborative groups – National Wilms Tumor Study Group (NWTSG)/Children's Oncology Group (COG) and The International Society of Paediatric Oncology (SIOP) have laid down the guidelines for standardized treatment of WT, though differing in the diagnostic and therapeutic approach. The major difference in the two guidelines is the timing of surgery: SIOP recommends using preoperative chemotherapy and NWTSG/COG prefers primary surgery before any adjuvant treatments. Both these groups currently aim at intensifying treatment for patients with poor prognosticators while appropriating the therapy to reduce long-term complications for those with favourable prognostic features. As the survival rate has now reached 90%, the primary objectives of the physician are to perform nephron-sparing surgery in selected cases and to reduce the dosage and duration of chemotherapy and radiotherapy in appropriate cases. The purpose of this review is to present current standards of diagnosis and treatment of WT around the world.

**Conclusion:**

Further studies in future should be done to highlight the use of chemotherapy and radiotherapy under risk-stratified strategies. Further improvement in survival of these children can only be achieved by increasing awareness, early recognition, appropriate referral, and a multidisciplinary approach.

## Highlights

• o Most of the patients with WT have good prognosis.
• o Multimodality treatment and multidisciplinary care are the major contributors for an improved prognosis.
• o Further studies should be done on usage of chemotherapy and radiotherapy under more accurate risk-stratified strategies and to decrease the late effects of surgery.

## 1 Introduction

Renal tumors are the fifth most common tumors in children and Wilms tumor (WT; nephroblastoma) is the most common paediatric renal tumor, accounting for about 85% of cases [1]. The incidence, growth rate, type and response to treatment of renal tumors in children differ significantly from adult renal cancers. Renal tumors in adults are mostly carcinomas, whereas in children they are of embryonic origin and thus they grow rapidly. Renal cell carcinomas, sarcomas and other tumors of the kidney are extremely rare in children. Moreover, childhood renal tumors have a better response to treatment as compared to adult tumors.

Wilms tumor was first reported by Thomas F. Rance in 1814. However, Max Wilms, a German surgeon and pathologist, gave the detailed description, adding seven new patients of his own in 1899 and since then the tumor bears his name [2]. It is primarily a disease of the kidney, but few rare extrarenal locations have been reported, like retroperitoneum, sacrococcygeal region, testis, uterus, inguinal canal and mediastinum [3].

Wilms tumor cells are believed to derive from pluripotent embyronic renal precursor cells. Thus, it is an embryonic renal tumor. While most are sporadic tumors, approximately 10% of cases are associated with genetic syndromes and extrarenal manifestations. There has been a dramatic improvement in overall survival rates due to, the coordinated use of modern surgical technique and anaesthesia, multiple drug chemotherapy and radiotherapy [4]. Large Mutidisciplinary cooperative cancer groups, namely the Children Oncology Group (COG) and the Société Internationale d’Oncologie Pédiatrique (SIOP) have laid down guidelines for standardized treatment of this entity and thus achieved a 5-year survival rate of more than 90%. This article reviews the genetics, imaging, histopathology and evolving treatment strategies of WT.

## 2 Epidemiology

Childhood cancers are uncommon, constituting 0.5–1% of all cancers, but are still the major cause of disease-related death in children. WT represents 6% of all childhood malignant tumors [5]. The incidence of WT is about 1 per 10,000 children in Europe and North America. There is a minor racial difference in the incidence of Wilms tumors. The Asian population has about half the incidence rate (3–4 per 10,000 children) of Western countries and its rate in the black population is 2.5 times higher [6]. The incidence of WT in other countries is similar to that in the USA. In Turkey, childhood renal tumors represent 7.1% of all childhood tumors [7]. The population-based incidence rate in a part of Italy was 4.5% for WT [8].

This tumor is seen mostly in children between the ages of 1 and 5 years and the peak age is 3. Although adult patients with WT have been reported, it is extremely rare in people older than 15 years of age [9]. COG revealed that the median onset age is 38 months and girls had disease onset 6 months later than boys. In most populations, no gender difference has been found; however, females are more likely to have WT than males (combined M:F = 1:4) in some Asian countries. For bilateral tumors, the median age at presentation is 29.5 months for males and 32.6 months for females. The male to female ratio is 0.92 for unilateral tumors and 0.6 for bilateral tumors. Most of the patients present before 5 years of age. WT is bilateral at presentation in 4%–8% of cases [10].

### 2.1 Molecular biology and genetics of Wilms tumor

Wilms tumor (hereditary or sporadic) appears to result from changes in one or more of several genes. WT1 and WT2 gene deletions are the two frequent genetic abnormalities in WT. A “two hit model” similar to that of retinoblastoma was proposed, indicating a recessive mutation in the etiology of this tumor. Apart from that, epigenetic alterations affecting 11p15 locus are associated with a selective increase in WT risk.

### 2.2 Genes and proteins involved

**WT1**: It is the first identified gene in WT and is responsible for development of genitourinary system. It is a tumor-suppressor gene located on chromosome 11p13. Its expression is seen in the kidney, gonads, spleen and mesothelium. It encodes four zinc finger transcriptional factors that have regulatory functions on cell growth, differentiation and apoptosis. Normal WT1 gene expression is necessary for the maturation of the blastemal cells and reduced WT1 expression is associated with the stromal predominant WT. It is deleted in WAGR and Denys-Drash syndrome [11].
**WT2**: This gene is located on chromosome 11p15 and is found in Beckwith-Wiedemann syndrome [12]. Some functions of this gene are related to insulin-like growth factor 2 (IGF2), which encodes embryonal growth factor.
**Other Genetic Abnormalities**: Other genes believed to be involved in WT development are, CTNNB1 (Beta-catenin), IGF2/H19, GPC3 (Glypican 3; Simpson-Golabi-Behmel gene). Another interesting observation is about Mulibrey nanism (for muscle-liver-brain-eye nanism, MUL). MUL is an autosomal recessive disorder that involves several tissues of mesodermal. About 4% of MUL patients develop Wilms tumor. There are rare inherited mutations. In addition to above genes, p57 Kip2 is also overexpressed or mutated in some patients. p57 Kip2 encodes cyclin-dependent kinase inhibitors and is a putative tumor suppressor [13].
**Beta-Catenin** is a cellular adhesion molecule that promotes overexpression of the c-myc and cyclin D1. Beta-Catenin mutations have been detected in 15% of patients with WT. There is a strong correlation between reduced expression of the WT1 gene and Beta-catenin mutations. Familial Wilms tumor has been found in 1–2% of cases. Although this tumor does have the WT1 gene, some familial tumors have linkage in the 17q, and this locus has been named FWT1. Some such tumors have demonstrated a 19q anomaly, which has been described as FWT2 [14].

The **p53**-encoded protein appears to act as a cell cycle checkpoint protein that arrests cell growth in G1. This gene regulates cell proliferation and induces apoptosis. Its inactivation results in genomic instability and cytogenetic aberrations (e.g. aneuploidy, translocations, deletions, and gene amplification). The Mutations of P53 occur in 5% of Wilms tumors and have been found in 75% of patients with anaplastic histology. TP53 abnormalities do not appear to associate with stage of (Diffuse anaplastic WT) DAWT but are associated with significantly worse disease-free and overall survival (OS) for patients with Stage III or IV DAWT. In addition to alterations at the TP53 locus, molecular profiling has demonstrated significant associations between anaplastic histology and loss of 4q and 14q [15].

Other chromosomal abnormalities, such as loss of heterozygosity (LOH) of 16q, 1p, and 7p, have been identified [14]. This defect has been associated with poor prognosis, relapses and death and has resulted in a poor outcome in patients with favourable histology WT. LOH at 1p and/or 16q associates with relapse and overall poor prognosis. Copy number gain of chromosome 1q is a commonly observed genetic abnormality in WT and is present in approximately 30% of tumors. After several smaller, retrospective studies suggested a correlation between 1q gain and tumor recurrence, the Children's Cancer and Leukaemia Group, NWTS, and SIOP independently confirmed poorer Event Free Survival and Overall Survival in both pre-treated and untreated patients with 1q gain in larger cohorts [14].

Genomic amplification of the MYCN gene has repeatedly been described in WT as well as other embryonal tumors, most commonly in neuroblastoma. Overexpression of MYCN in WT has been identified as a potentially prognostic feature. Interestingly, MYCN gain was present in higher proportion (>30%) among a cohort of pre-treated anaplastic tumors compared with a parallel study analyzing a mixed cohort of anaplastic tumors (which included tumors that were not pre-treated). This suggests that MYCN gain could confer treatment resistance. Notably, MYCN gain is not limited to anaplastic WT, and its association with poorer relapse-free and overall survival is independent of histology. The P44L mutation has been identified as a potentially activating mutation leading to MYCN gain in WT [14].

Frequency of LOH at 11q was 3–4 times higher among mixed type and diffuse anaplastic tumors compared to favourable histology tumors. Loss of the entire long arm of chromosome 11 was associated with higher rates of relapse and death. Other studies have also demonstrated a correlation between LOH at 11q and anaplasia, tumor recurrence, and death, indicating that this region is likely prognostically relevant [14].

### 2.3 DNA content

Some studies suggested that flow cytometric evaluation of DNA-ploidy is a useful predictor of outcome and response to therapy. Diploid and aneuploid tumors are reported to have better long-term survival when compared with tetraploid tumors. However, other studies reported that this factor is not superior compared to histology and staging. Ongoing studies will determine the clinical usefulness of DNA Ploidy [16].

### 2.4 Hereditary factors

Despite the number of genes that appear to be involved in the development of WT, hereditary WT (either bilateral tumors or a family history of the neoplasm) is uncommon, 1%–2% of patients having a positive family history for WT. The risk of WT among offspring of persons who have had unilateral (i.e., sporadic) tumors is quite low (<2%). Siblings of children with WT have a low likelihood of developing Wilms tumor. A second WT may develop in the remaining kidney of 1%–3% of children treated successfully for Wilms tumor. The incidence of such metachronous bilateral Wilms tumors is much higher in children whose original WT was diagnosed at less than 12 months of age and/or whose resected kidney contains nephrogenic rests. Periodic abdominal ultrasound is recommended for early detection of metachronous bilateral WT as follows: children with nephrogenic rests in the resected kidney (if < 48 months of age at initial diagnosis) - every 3 months for 6 years; children with nephrogenic rests in the resected kidney (if > 48 months of age at initial diagnosis) - every 3 months for 4 years; other patients - every 6 months for 2 years, then yearly for an additional 1–3 years [16].

### 2.5 Associated congenital anomalies

In 10%–13% of cases, WT is associated with several congenital anomalies. Children with genitourinary anomalies such as horseshoe kidney, renal dysplasia, bilateral cystic renal disease, double collecting system, fused kidney, cryptorchidism, hypospadias, aniridia and hemihypertrophy have a higher incidence of WT [17]. Congenital abnormalities are seen more commonly in bilateral tumors. In addition, it is a component of the syndromes described below.

(a) **Beckwith-Wiedemann Syndrome:** This syndrome is associated with macroglossia, visceromegaly, omphalocele and gigantism. About 4–5% of patients with this syndrome have WT. The molecular defect is on chromosome 11p15.5 [12]. IGF-2 abnormalities are related to this gene and may be responsible for the development of Wilms tumor and the Beckwith-Wiedemann syndrome.
(b) **WAGR Syndrome:** The components of this syndrome are WT, aniridia, genitourinary abnormalities and mental retardation. Cardiopulmonary problems, head anomalies, neurobehavioral disorders, musculoskeletal defects and metabolic problems have also been reported [11]. The 11p13 chromosomal deletion has been identified. The Wilms tumor risk is 30% in this syndrome.
(c) **Denys-Drash Syndrome**: It is a combination of Male pseudohermaphroditism, glomerulonephritis and WT. There is also an association with a defect on the WT1 gene [17].
(d) **Perlman Syndrome**: This syndrome can be associated with WT and includes macrosomia, islet cell hyperplasia, renal hamartomas and an atypical face shape [17].

The other associations of WT are 13 and 18 trisomies [18], cerebral gigantism and neurofibromatosis. Other associated malformations include Septal defects, microcephalus, hyperinsulinism and von Willebrand's disease (8%). WT is rarely associated with metastasis at time of diagnosis. The most common site of metastasis is lung (85% of cases), followed by the liver and regional lymph nodes [17,18].

## 3 Recent advances in WT predisposition

Recently, several reports have described novel genes in which pathogenic germline variants confer an increased risk for WT, including CTR9, REST and TRIM28.

CTR9 encodes a component of the Polymerase-Associated Factor 1 (PAF1) complex, which associates with RNA polymerase II, a large protein complex that transcribes DNA into messenger RNA and several small nuclear RNAs. The human PAF1 complex is comprised of several subunits including CTR9, CDC73, LEO1, PAF1, RTF1 and SKI8. Studies of the Paf1 complex in yeast have revealed multiple roles including gene regulation, transcriptional elongation and chromatin modifications. To date, four unrelated families harboring germline variants in CTR9 have been reported. Descriptions of the WTs developing in CTR9 families are somewhat limited. Although the exact mechanism by which CTR9 LOF contributes to kidney tumor formation is yet to be determined, as the PAF1 complex is involved in regulation of gene expression, DNA repair and cell cycle, it is possible that a disturbance in one or more of these functions leads to Wilms’ tumorigenesis.

RE1-silencing transcription factor (REST; also known as Neuron Restrictive Silencer Factor) encodes a Krüppel-associated box (KRAB) zinc-finger transcription factor made up of two repressor domains (RD1, RD2) and a DNA-binding domain (DBD). REST serves as a focal point for the recruitment of chromatin modifying enzymes that silence the expression of target genes and play a critical role during embryonic development and neurogenesis. While the mechanism(s) by which gremlin variants in REST contribute to WT remain to be determined some KRAB zinc-finger proteins can mediate transcriptional repression by recruiting the c regulator TRIM28, a gene in which pathogenic germline variants also predispose to WT.

Tripartite motif containing 28 (TRIM28; also known as KAP1 serves as a co-regulator for the KRAB proteins. TRIM28-associated complexes contribute to many aspects of cellular biology, including proliferation, genome stability, immune response, early embryonic development and embryonic stem cell pluripotency. Importantly, TRIM28 controls genomic imprinting through distinct mechanisms at different developmental stages. TRIM28-associated WT often contains a predominance of epithelial cells, which generally express lower levels of IGF2. Thus, IGF2 upregulation may not be as crucial for the formation of epithelial-predominant TRIM28-associated tumors. This is further supported by reports of lower IGF2 expression and normal imprinting of 11p15 in a subset of tumors exhibiting epithelial-predominant histology. TRIM28 functions as a classical tumor suppressor gene. While the mechanisms by which TRIM28 inactivation induces Wilms tumorigenesis remain to be elucidated, TRIM28 plays an important role in the developing kidney [19].

Evaluation and management of children with hereditary predisposition to WT.

In the absence of syndromic features or a family history suggestive of any specific cancer predisposition syndromes, clinical germline testing should include sequencing and deletion/duplication analysis of CTR9, DICER1, REST, TP53, TRIM28 and WT1; other genes can be added at the discretion of the genetics provider, based on personal medical or family history features. If the child is found to harbor a pathogenic or likely pathogenic variant in a WT predisposition gene, his or her parents and close relatives can also be offered testing. Affected individuals should be counseled about the risks for additional neoplasms and non oncologic manifestations, as appropriate, as well as the risks for recurrence in future offspring.

Children testing positive should be offered surveillance throughout the period of increased WT risk, which is typically up to 8 years of age but may vary depending upon the condition. The goal of surveillance is to detectWTs while they are low stage and more likely to be cured using fewer intensive therapies. Luckily, it is easy to visualize the kidneys by radiologic methods such as ultrasound, which is a readily available, safe and easy and relatively inexpensive procedure. As WTs can double in size every week, it is recommended that an abdominal ultrasound be completed once every 3 months. Additional modes of surveillance can be considered for individuals with predisposition to a wider spectrum of cancers, such as those with Li-Fraumeni or DICER1 syndrome.

**Fig. 1:**
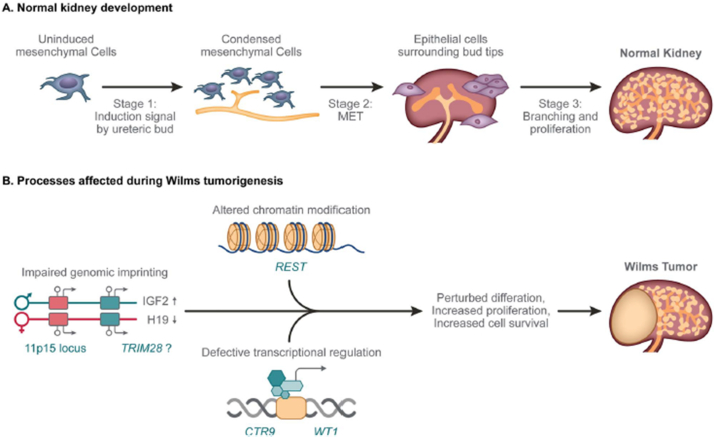
Normal kidney development and processes affected by WT predisposing lesions. (A) During normal kidney development, mesenchymal cells condense around the ureteric bud tips (Stage 1) to form an aggregate that undergoes a MET (Stage 2), resulting in the formation of nephrons, and after branching and proliferation (Stage 3), a healthy kidney. (B) WT predisposing lesions alter genomic imprinting (11p15, TRIM28), chromatin modification (REST) and transcriptional regulation (CTR9, WT1), leading to perturbations in normal kidney differentiation and generation of a cellular state that is poised for malignant transformation upon acquisition of cooperating somatic genetic lesions.

Genetic and/or epigenetic discoveries may be challenging as causal lesions could reside in non-coding regions of the genome or involve more complex mechanisms, such as structural variants (including intronic deletions or inversions), digenic or polygenic inheritance and events that occur post zygotically [19] (see Fig. 1).

### 3.1 Clinical presentation

**Fig. 2:**
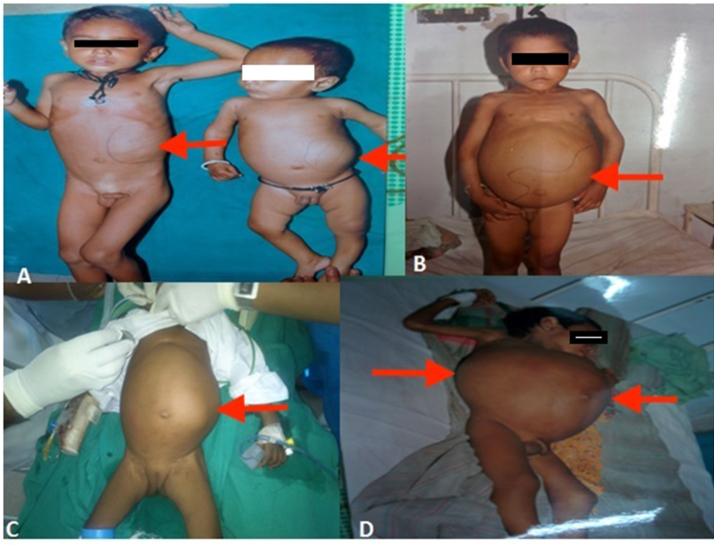
Clinical photograph showing different patients presenting with an abdominal lump.

Most of the patients present with an abdominal mass (Fig. 2). The tumor is often detected by the parents or caregivers while bathing the child. Haematuria is seen in 30% of patients and 25% have hypertension. In addition, malaise, fever, weight loss, anorexia, or a combination of these symptoms can be seen. The tumor can rupture with trivial trauma and these patients present with acute abdominal pain. Obstruction of the left spermatic vein by the mass can result in a left-sided varicocele. Few hormones, such as erythropoietin and ACTH, can be secreted in WT. In addition, hypercalcemia and haemorrhagic conditions caused by reduced von Willebrand factor can be seen [20]. Physicians must be cautious for other associated findings, such as hemihypertrophy, aniridia and genitourinary malformations.

### 3.2 Laboratory tests

Laboratory tests are done to check urine and blood samples if a kidney problem is suspected. They may also be done after a WT has been found. A urine sample may be tested (urinalysis) to see if there are problems with the kidneys. Urine may also be tested for substances called catecholamines. This is done to make sure the child doesn't have another kind of tumor called neuroblastoma. So, the battery of tests usually employed includes Complete Blood Counts, coagulation Profile, Urine routine, microscopy and culture.

Certain laboratory tests are important for proper management of WT cases. Recently, urinary basic Fibroblast Growth Factor (bFGF) has been reported to be elevated preoperatively in these patients [16]. The serum level of neuron-specific enolase (NSE) and urinary catecholamine levels should be routinely measured to exclude neuroblastoma, which is a very close differential of WT. Tissue Polypeptide Specific antigen (TPS), might be of clinical value in monitoring the therapy of WT [21].

### 3.3 Radiological investigations

Advances in radiological techniques are able to detect non-palpable WT and its spread much earlier than in the past. Before the ultrasonography (USG) and tomography era, direct radiograph and intravenous urography were used widely. Ultrasound is commonly used for the initial evaluation of renal tumors, and imaging features associated with a renal origin include a mass that moves with respiration. On ultrasound, WT commonly presents as an echogenic mass with discrete hypoechoic areas corresponding to necrosis. Calyceal distortion with renal displacement is the characteristic finding “claw sign” (Fig. 3A). Few cases of WT have been diagnosed antenatally with help of USG. It is usually associated with polyhydramnios. Increased mortality has been reported if associated with fetal hydrops. Doppler USG shows vena cava invasion, which is important for determining the preoperative treatment strategy. USG and contrast-enhanced computed tomography (CECT) of the abdomen are more effective diagnostic techniques in the staging and follow-up of patients, as they can detect tumor size, invasion and tumoral involvement of the lymph nodes.

**Fig. 3A:**
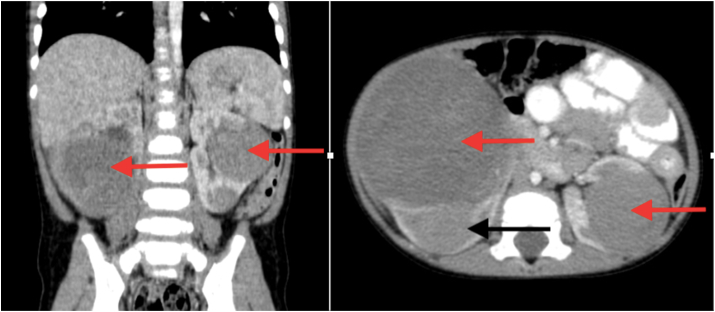
Axial CT image from a patient with Wilms tumor showing the characteristic “claw sign” seen in tumors of renal origin, in which the enhancing renal parenchyma is displaced by the enlarging tumor.

While US is a useful starting point for imaging, all paediatric patients with renal masses should undergo cross-sectional imaging with computed tomography (CT) or magnetic resonance imaging (MRI), as in over half of patients these studies provide additional important information beyond what can be obtained with US. CT shows other parenchymal organ metastasis, such as to the liver, the extent of renal involvement including the contralateral kidney (Fig. 3B), the renal vein, and the inferior vena cava (IVC). These important features are critical for accurate staging, as preoperative identification of any of the above findings can affect staging and treatment assignment. Skiagram chest, CT scans of the chest and the abdomen should also be done as baseline diagnostic procedures for complete evaluation of the extent of the mass and distant spread if any. MRI studies have a predominant role in demonstrating the relation of the tumor to other organs. MRI is more sensitive than CT scan. Nephrogenic rests appear as small homogeneous lesions after Gadolinium enhancement, different from the heterogeneous appearance of WT. Tumor calcifications, when present in WT, mean that tumor growth is slow, and possibly a good prognostic sign [22]. Contrasted CT or MRI can provide even more definitive information about resectability of tumors and the presence of intravascular tumor, which occurs in 6% of Wilms tumor patients. Because vascular extension of tumor greatly increases the surgical complication rate, upfront nephrectomy is usually deferred while patients are treated with neoadjuvant chemotherapy in an attempt to retract the clot and facilitate a safer surgery done later. MRI is recommended by the Children's Oncology Group for evaluation of patients with known or suspected bilateral Wilms tumor.

**Fig. 3B:**
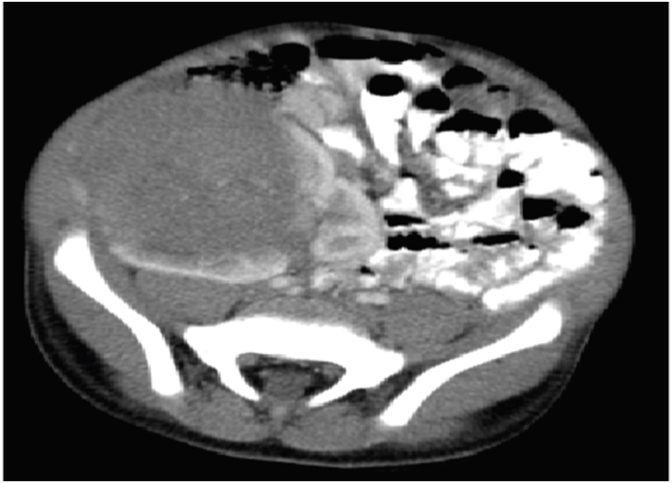
Computed tomography (CT) scan image of abdomen (coronal and transverse sections) showing tumors (red arrow) involving bilateral kidneys and compressed normal part of left kidney (black arrow). (For interpretation of the references to color in this figure legend, the reader is referred to the Web version of this article.)

The lungs are the most common site of metastasis in Wilms tumor, and historically patients were considered to have lung involvement if nodules were identified on chest X-ray (CXR). However, CT of the chest is a more sensitive modality for identifying metastatic lung nodules, especially when done preoperatively in awake patient in order to reduce the atelectasis associated with postoperative or sedated imaging studies in young children. Five percent of Wilms tumor patients will have nodules identified only on CT but not conventional CXR. The use of diffusion weighted MRI has been reported to show correlations between the apparent diffusion coefficient measurements and the blastemal component of residual tumors after neoadjuvant chemotherapy.

Finally, surveillance imaging is also utilized in pre Cancerous patients who remain at high risk for the development of WT. Given the known association with a variety of cancer predisposition syndromes, guidelines have been developed for following these at-risk patients in a way that balances the benefits of early identification with safe and reasonable utilization of imaging. Specifically, renal USs are recommended every 3 months from the time the predisposition syndrome is diagnosed at least until the 7th birthday [23].

WT can be radiologicaly differentiated from neuroblastoma, which is a close mimicker (Table 1). Invasion of the inferior vena cava, which can occasionally extend to the right atrium is strongly predictive of WT, while paravertebral mass with spinal canal invasion is for neuroblastoma. The spread around the celiac and superior mesenteric arteries differentiates neuroblastoma from WT, as is most common in the neuroblastoma. If there is no clear discrimination from neuroblastoma, an I-metaiodobenzylguanidine (MIBG)-scan may be performed. In renal tumors, monitoring ultrasound before and after the treatment, must be performed periodically every three months. Even after nephrectomy of the affected kidney, the other kidney monitoring should be performed [23].

**Table 1:** Comparison of radiological features of Wilms Tumour and Neuroblastoma.

	WILMS TUMOUR	NEUROBLASTOMA
**X-RAY**	Outlines tumour mass with adjacent structures displacement and lifting the diaphragm side of the lesion, and may occasionally present calcifications.	Large abdominal mass with thin and dotted calcifications.
**ULTRASOUND**	Large solid mass with Homogeneous echogenicity, well-defined, with and discrete hypoechoic areas corresponding to necrosis, causing distortion and displacement of the collector system and the capsule.	Solid adrenal heterogeneous mass, poorly defined and may present calcifications, necrosis and haemorrhage.
**CT SCAN**	Spherical intrarenal mass, heterogeneous with calcifications and fat. Well defined, with mixed attenuation, little contrast enhancement, cystic areas due to haemorrhage and necrosis, and lymph node metastasis.	Poorly defined mass that
	When originates from cortex can have exophytic growth.	May have calcifications- occupying most of the central abdomen.
	Used for staging.	There is engagement/displacement of the great vessels, renal vessels and extending to periaortic and retro-crural lymph nodes.
**MRI**	Well-defined mass with relatively distinct margins with predominantly low signal on T1-weighted images and high signal on T2-weighted images. Often appears heterogeneous in T1 and T2 weight images.	Good for viewing the involvement of the spinal canal, being seen as foci of low signal intensity on T1 weighted images.
	Demonstrates better venous extension CT scan.	
	Used for staging.	

### 3.4 Gross

**Fig. 4:**
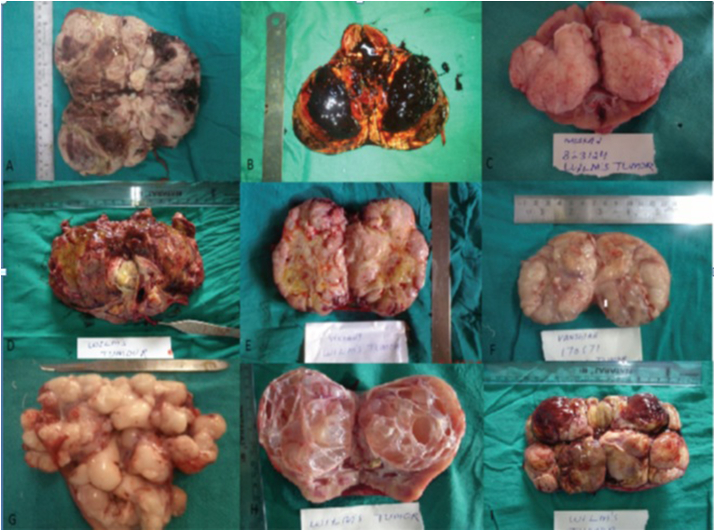
Photograph of different cut open nephrectomy specimens showing various patterns of tumor on gross examination: (A) haemorrhage and necrosis, (B) haemorrhage, (C) organoid, (D) rhabdoid, (E) fish flesh appearance, (F) lobular, (G) sarcoma botyroides pattern, (H) cystic and (I) teratoma appearance.

The usual gross appearance of WT is a large, solitary, well circumscribed mass (10% bilateral or multicentric) that is soft, homogenous and tan grey in color. Haemorrhage, necrosis, cysts and lobular pattern are common. But gross appearances may vary (Fig. 4).

### 3.5 Histopathology

**Fig. 5:**
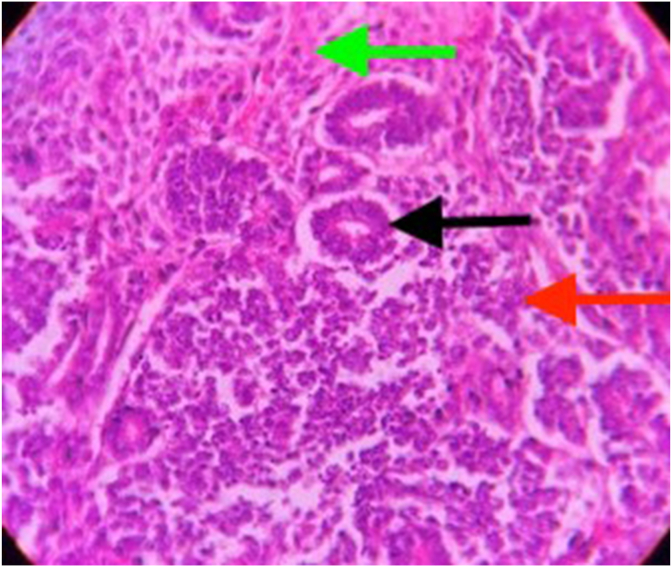
Photomicrograph (100x, H&E) showing histological features of a classical Wilms tumor having all the three components: blastemal (red arrow), epithelial (black arrow) and stromal (green arrow). (For interpretation of the references to color in this figure legend, the reader is referred to the Web version of this article.)

There is mimicry of nephrogenesis in WT as the tumor comprises of 3 elements namely, undifferentiated blastemal cells, differentiated epithelial cells and stromal cells. Ectopic components like skeletal muscle may be observed in 5–10% of tumors. The stromal components are believed to be neoplastic, raising the possibility that undifferentiated blastema cells are precursors of the stromal and heterologous elements. There are two main histological types of WT [24]:

(a) **Classical nephroblastoma**:

This entity includes blastemal, epithelial and stromal components. Sometimes one or two components are predominant, and sometimes they are equally present. The latter type of tumor is classified as a mixed-type or triphasic Wilms tumor (Fig. 5).

(b) **Anaplastic Wilms tumor:**

There are three main cytopathologic features of anaplasia: a) a threefold or greater nuclear enlargement, compared to the nearby nuclei of the same cell type e.g. stromal or epithelial; b) hyperchromatism (indicating that the nuclear enlargement is attributable to gross polyploidy and not to hydrophilic swelling or poor fixation) and c) enlarged abnormal (usually multipolar) mitotic figures, which is regarded as the most quintessential criterion. It constitutes 4–8% of all cases. This type may have a diffuse or focal form and this classification has prognostic importance, as patients with focal anaplasia should be treated with less intensive protocols than those with diffuse anaplasia. Previously, a tumor was classified as focal anaplastic, if anaplastic cells were encountered in fewer than 10% of microscopic fields. This description was revised by Faria et al. [25] in 1996, as follows: In focal anaplasia, anaplastic changes are confined to circumscribed regions within the primary tumor and are surrounded by non-anaplastic tissue. Diffuse anaplasia has the following characteristics: it is found in an extrarenal site, the random biopsy specimen reveals unequivocal anaplasia, the tumor is coupled with extreme nuclear unrest, and there is nuclear atypia elsewhere in the tumor [25]. This classification of focal and diffuse anaplasia has been used in the COG; the other large-scale collaborative group, the SIOP, stratifies risk groups according to histopathologic structures.

SIOP has analyzed risk for two groups: those patients who have been pre-treated and those receiving primary nephrectomy. Table 2 shows SIOP risk groups according to histopathology [25]. Tumor classification should determine the choice of treatment protocols. Anaplastic tumors, except for those in stage 1, should be treated with more intensive protocols than mixed-type tumors [26].

**Nephrogenic Rests:** The nephrogenic rest (NR) is the putative precursor lesion of the WT, which sometimes can be confused with malignancies and include blastemal, stromal and embryonal elements. It can be found in the opposite or the same foci in the affected kidney. If located peripherally, it is classified as a perilobar nephrogenic rest; if located deep in the renal lobe, it is an intralobar nephrogenic rest. They can regress or stay dormant [24]. NRs may be microscopic or grossly visible, single or multiple. Patients with NRs (in particular perilobar NRs), have a significantly increased risk of metachronous bilateral Wilms tumor.

**Table 2:** Revised SIOP working classification of renal tumors of childhood.

A.	For pretreated cases	
a.	Low-risk tumors	Congenital Mesoblastic nephroma
		Cystic partially differentiated nephroblastoma
		Completely necrotic nephroblastoma
b.	Intermediate-risk tumors	Nephroblastoma-epithelial type
		Nephroblastoma-stromal type
		Nephroblastoma-mixed type
		Nephroblastoma-regressive type
		Nephroblastoma-focal anaplasia
c.	High-risk tumors	Nephroblastoma-blastemal type
		Nephroblastoma-diffuse anaplasia
		Clear cell sarcoma of the kidney
		Rhabdoid tumour of the kidney
		Renal Cell carcinoma
B.	**For primary nephrectomy cases**	
a.	Low-risk tumors	Congenital Mesoblastic nephroma Cystic partially differentiated nephroblastoma
b.	Intermediate-risk tumors	Non-anaplastic nephroblastoma and its variants
		Nephroblastoma-focal anaplasia
c.	High-risk tumors	Nephroblastoma-diffuse anaplasia
		Clear cell sarcoma of the kidney
		Rhabdoid tumour of the kidney
		Renal Cell carcinoma

Although most patients with a histologic diagnosis of Wilms tumor fare well with current treatment, approximately 12% of patients have histopathologic features that are associated with a poorer prognosis, and, in some types, with a high incidence of relapse and death. WT can be separated into 2 prognostic groups on the basis of histopathology:

(a) Favourable histology:

Histology mimics development of a normal kidney consisting of 3 components: blastema, epithelium (tubules) and stroma. There is no anaplasia. This Corresponds to Favourable Histology.

(b) Unfavourable histology:

Characterized by anaplasia. Focal anaplasia may not confer nearly as poor prognosis as diffuse anaplasia. Anaplasia is associated with resistance to chemotherapy and may still be detected after pre-operative chemotherapy. While this group corresponds to Unfavourable Histology.

### 3.6 Staging system for Wilms tumor

There is involvement of two large groups in management of WT: the Children Oncology Group (COG) and the Société Internationale d’Oncologie Pédiatrique (SIOP). They use similar staging systems with only minor differences. SIOP gives preoperative chemotherapy and then does staging after preoperative treatment and surgery. The COG group treats patients with surgery at the time of diagnosis, and then they are staged. The staging system of these two groups is shown in Table 3 [26,27]. Each side must be staged individually according to the criteria mentioned above. Therapy would be offered based on the higher stage of the two.

**Table 3:** Staging system for Wilms tumour.

STAGE	COG	SIOP
**I**	Tumour is limited to kidney and completely resected.	Tumour is limited to kidney and completely resected.
	Renal capsule intact; not penetrated by tumor.	Tumor is present in the perirenal fat but is surrounded by a fibrous (pseudo)capsule; the (pseudo)capsule might be infiltrated by viable tumor, which does not reach the outer surface. Tumor might show protruding (‘botryoid’) growth into the renal pelvis or ureter but does not infiltrate their walls.
	Tumor not ruptured or biopsied prior to removal.	The vessels or the soft tissues of the renal sinus and not involved by tumor; intrarenal vessel involvement might be present.
	No tumor invasion of veins or lymphatics of renal sinus	
	No nodal or hematogenous metastases.	
	No rupture or biopsy prior to removal.	
**II**	Tumor in the perirenal fat but completely resected.	Viable tumor is present in the perirenal fat and is not covered by a (pseudo)capsule but is completely resected (resection margins are clear).
	Tumor infiltrates the renal sinus or blood and lymphatic vessels outside the renal parenchyma but is completely resected.	Viable tumor infiltrates the soft tissues of the renal sinus.
	Tumor infiltrates adjacent organs or vena cava but is completely resected.	Viable tumor infiltrates blood or lymphatic vessels of the renal sinus or of the perirenal tissue.
		Viable tumor infiltrates the wall of the renal pelvis or the ureter.
		Viable tumor infiltrates the vena cava or adjacent organs (except the adrenal gland) but is completely resected.
**III**	Residual tumor or nonhematogenous metastases confined to abdomen.	Viable tumor present at resection margin(s).
	Involved abdominal lymph nodes.	Abdominal lymph nodes contain viable or nonviable tumor.
	Peritoneal tumor implants.	Viable or nonviable thrombus present at resection margins of ureter, renal vein or inferior vena cava.
	Tumor spillage before or during surgery.	Viable or nonviable tumor thrombus in the inferior vena cava removed piecemeal by a surgeon.
	Gross residual tumor in abdomen.	Preoperative or intraoperative tumor rupture, if confirmed by microscopic examination (viable tumor at the surface of the specimen at the area of rupture).
	Biopsy of tumor (including fine needle biopsy) prior to removal of kidney.	Wedge or open biopsy before preoperative chemotherapy or surgery.
	Resection margins involved by tumor or transection of tumor during resection (i.e. piecemeal excision of tumor).	Tumor implants (viable or nonviable) in the abdomen.
		Tumor (viable or nonviable) penetrated through the peritoneal surface.
**IV**	Hematogenous metastases or spreads beyond abdomen.	Hematogenous metastases (lung, liver, bone, brain) or lymph node metastases outside the abdominopelvic region.
**V**	Bilateral tumors at diagnosis; each side should be substaged according to the above criteria.	Bilateral tumors at diagnosis; each side should be substaged according to the above criteria.

### 3.7 Wilms Tumor in adults

WT is the most common abdominal tumor in children; but it is extremely rare in adults, representing only 0.5% of all renal neoplasms. Till date only 240 cases in adults have been reported in the literature [28]. The diagnostic criteria defining adult WT were described by Kilton et al. [29]. It is difficult to differentiate this entity from renal cell carcinoma based only on imaging techniques, though preoperative diagnosis may be suggestive in about 75–80% of cases. On USG, it presents as a rapidly growing abdominal mass, with heterogeneous contrast uptake, and is surrounded by a pseudocapsule on CT which is suggestive of WT. Arteriography characteristically shows a hypovascular mass with neo-formed blood vessels exhibiting a zigzag pattern. The histopathological study confirms its diagnosis. The treatment is not well established for adults. Aggressive treatment, including radical surgery, chemotherapy and irradiation of the tumor bed, is considered necessary. The chemotherapeutic agents routinely used are vincristine, actinomycin-D, doxorubicin and ifosfamide. Satisfactory results have also been obtained with cisplatin and etoposide in patients with stage IV disease and patients in progression after conventional chemotherapy. The prognosis in adults is worse than in children. This may be due to the fact that, adults do not receive paediatric protocols, as has been demonstrated [30].

### 3.8 Treatment

Multidisciplinary approach is required to determine and implement optimum treatment for WT. Ideal team includes experienced paediatric surgeon or paediatric urologist, paediatric radiation oncologist and paediatric oncologist. COG and SIOP guidelines provide two different strategies for the treatment of WT in children. COG recommends patients undergo surgery before chemotherapy. North America commonly adopts COG guideline. However, most children in European countries are treated with preoperative chemotherapy based on the SIOP guideline. Different treatment strategies are based on different staging systems. The COG staging system relies on pathological analysis from a primary nephrectomy in most cases. The SIOP staging is based on the results after preoperative chemotherapy. But the overall survival rate for the patients treated by the two guidelines is almost similar i.e. approximately 90% [31]. Multimodality Therapy consists of surgery and chemotherapy, with radiation or those who also need it. Its components are described as below.

(a) Surgery

**Fig. 6:**
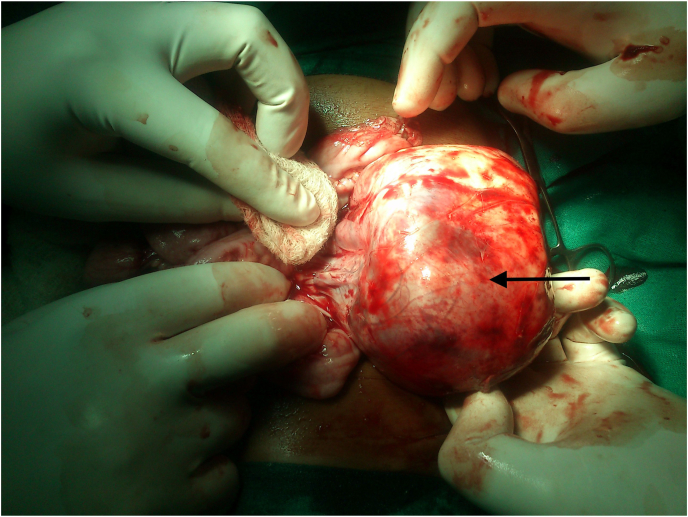
Intra-operative photograph showing an operable well encapsulated Wilms tumor involving left kidney for contemplated en bloc resection.

Surgery is the cornerstone of the treatment. Operative principles have evolved from COG trials (Table 4). The crucial role of surgeon is to ensure complete removal of the tumor without rupture and perform an assessment of the extent of disease (Fig. 6). Radical nephrectomy via a transabdominal incision and lymph node sampling is the procedure of choice. Transperitoneal approaches are used, as the flank incision is not suitable for WT because there is increased risk of spill over of tumor and moreover, access to lymph nodes is harder. Hilar, peri-aortic and iliac lymph node sampling is must. Lymph node sampling is important for staging. Furthermore, any suspicious node should be sampled. Margins of resection, residual tumor and any suspicious node basins should be marked with titanium clips. Titanium clips are specifically not to be used per COG protocols unless there is gross residual disease left in the abdomen. Pre-operative chemotherapy does not make the resection easier. It may shrink a thrombus or spare organs to allow resection, but it does not make the surgery easier. In fact it is exactly opposite in unilateral tumors, it obliterates planes and makes the case much more difficult. The SIOP recommends radical tumor nephrectomy performed after preoperative chemotherapy. Patients had minimal complications and no increased risk of local recurrence or upstaging [26]. Ligation of both the renal artery and vein is preferable before performing radical nephrectomy. WT is an encapsulated tumor (Fig. 6), and en bloc resection can be done to avoid tumor spillage. Resection of the primary renal tumor should be considered even if in a stage IV disease (usually pulmonary metastases). The incidence of post-operative complications in the COGG was 11%. The most serious complication intraoperatively is tumor embolus into pulmonary artery and sudden death. Common post-operative complications are haemorrhage and intestinal obstruction. Intestinal obstruction in first post-operative week is mostly due to intussusception and after that is due to adhesive obstruction.

**Table 4:** Surgical Principles used for Wilms tumour.

A.	STANDARD PROCEDURE	Radical nephrectomy + lymph node sampling through a transperitoneal approach
		Surgery helps in - Assessing Tumour extent involvement
		-Lymph node sampling
		-Any liver metastasis biopsy
		-Any peritoneal seeding biopsy
**B.**	**ROLE OF CONTRALATERAL EXPLORATION**	•With the availability of modern high-quality cross-sectional imaging, contralateral renal exploration for patients undergoing surgery for unilateral WT is largely unnecessary.
		•Historically, contralateral exploration was recommended when excretory urography was the only pre-operative imaging modality.
		•Now, with the advancement of CT scan and MRI, lesions measuring millimeters can be detected pre-operatively.
**C.**	**INTRAVASCULAR TUMOUR EXTENSION**	•Neoadjuvant chemotherapy is helpful in avoiding a tumour embolus during mobilization.
		•An infradiaphragmatic infrahepatic non-adherent caval vein thrombus generally can be removed by cavotomy or using a Fogarty or Foley balloon catheter.
		•Patients with intravascular extension above the level of the hepatic veins should receive preoperative chemotherapy.
		•Recent reports show that preoperative therapy in patients with suprahepatic caval or atrial extension led to a marked decrease in size of tumour thrombus and even complete regression of thrombus without embolization.
		•As an alternative in adverse cases, embolectomy under cardiopulmonary bypass is required.
**D.**	**PARENCHYMAL SPARING SURGERY**	Partial nephrectomy
		Enucleation
		The above procedures can be done if following criteria are satisfied:
		a.Tumour involving one pole and less than one-third of kidney
		b. Normal functioning remaining kidney
		c.No tumour extension into renal collecting system and renal vein
		d. Clear demarcation between tumour and kidney and adjacent structures
		**INDICATIONS:**
		•Bilateral WT
		•Renal insufficiency as in Denys-Drash syndrome
		•Solitary kidney WT
		•Syndromes associated with increased incidence of nephrogenic rests.

**ROLE OF CONTRALATERAL EXPLORATION:** With the availability of modern high-quality cross-sectional imaging, contralateral renal exploration for patients undergoing surgery for unilateral WT is largely unnecessary. Historically, contralateral exploration was recommended when excretory urography was the only pre-operative imaging modality. Now, with the advancement of CT scan and MRI, lesions measuring millimeters can be detected pre-operatively. Several studies have demonstrated high sensitivity and specificity (close to 100%) with these modalities, with no evidence of missed disease during contralateral exploration. Ritchey et al. found that routine contralateral exploration may yield a small number of occult lesions not identified on pre-operative imaging, but that omission of routine contralateral exploration is unlikely to affect the outcome of any children with newly diagnosed WT, as long as they underwent CT or MRI scan prior to surgery [32].

(b) Partial nephrectomy

The role of partial nephrectomy (nephron-sparing surgery) remains controversial. This surgery is not recommended by COG guidelines, except when children have a solitary kidney, with predisposition to bilateral tumors, horseshoe kidney or in infants with Denys-Drash or Frasier syndrome (to delay the need for dialysis). This is a SIOP recommendation only. It is used for non-syndromic unilateral WTs with small tumor volume (<300 mL) and the expectation of a substantial remnant kidney function in patients who never had lymph node involvement. Several studies reported an increased incidence of hypertension, proteinuria and decreased renal function, even renal failure, in patients who underwent unilateral nephrectomy for WT. Total tumor nephrectomy might potentially be harmful to the patient due the substantial risk of renal function loss of a solitary kidney caused by the consecutive hypertrophy of the remaining contralateral kidney as well as to the probability of a primary malformation, metachronous tumor occurrence (1.5% in COG, 2–3% in SIOP studies), accidental damage, or other superimposed renal injury. The currently reported poor evidence of a marked risk of renal failure following unilateral nephrectomy however might be due to the lack of long-term follow-up studies. Surgical (radiological and pathological) selection criteria for partial nephrectomy should include functioning kidney, tumor confined to one pole occupying less than one third of the kidney, no invasion of the renal vein or collecting system, and clear margins between tumor, kidney, and surrounding structures. Most studies concur that safe partial nephrectomy is applicable in approximately 5% of tumors at diagnosis (10% of patients after preoperative chemotherapy) without violating oncological principles. The local recurrence rate for partial nephrectomy in patients with bilateral tumors was found to be 8.2% [33].

(c) Chemotherapy

Chemotherapy has proved to be beneficial in all stages of the disease and radiotherapy is used to improve the outcome of late stage tumors, including stage II malignancies with diffuse anaplasia. Chemotherapy can be given prior to surgery or after surgery (Table 5).

**Table 5:** Guidelines for chemotherapy in Wilms tumour.

NEOADJUVANT CHEMOTHERAPY	
Indications:	
•Bilateral Wilms tumour	
•Inoperable tumour	
•Intravascular extension into IVC above hepatic veins	
•Tumour in solitary kidney	
•Chronic Kidney Disease	
•Predisposition syndromes	
PREOPERATIVE CHEMOTHERAPY [7,8]	
**COG GUIDELINES**	**SIOP GUIDELINES**
•The COG guideline recommends surgery as the initial therapy before chemotherapy.	•The SIOP guideline recommends preoperative chemotherapy for all patients after diagnosis.
•INDICATIONS: with inoperable WT; with a solitary kidney; with synchronous bilateral WT; tumour thrombus in the inferior vena cava extending above the level of the hepatic veins; tumour involving contiguous structures whereby removing the kidney tumour requiring removal of the other organs, such as spleen, pancreas, or colon and with extensive pulmonary metastases [16].	•For patients with unilateral localized tumour, 4-week pretreatment with vincristine (weekly) and dactinomycin (biweekly) is given.
•The agents for chemotherapy commonly are doxorubicin plus dactinomycin and vincristine; if with anaplastic histology, chemotherapy then includes regimen I.	•For patients with bilateral tumors, vincristine– dactinomycin for no longer than 9–12 weeks is recommended (doxorubicin is added for reinforcement in some patients).
	•For patients with metastasis, a regimen including 6 weeks of vincristine–dactinomycin (like above) and doxorubicine on weeks 1 and 5 is given.
**POSTOPERATIVE CHEMOTHERAPY**	
**COG GUIDELINES**	**SIOP GUIDELINES**
•The COG recommends postoperative chemotherapy routinely used in all patients with WT except those at a very low risk: younger than 2 years at diagnosis with stage I favourable histology tumour weighing <550 g was sampled and confirmed negative lymph nodes.	•The SIOP recommends postoperative chemotherapy in all patients with WT except those with stage I low risk tumour.

**COG Studies:** The COG guideline recommends surgery as the initial therapy before chemotherapy. Preoperative chemotherapy is only indicated under the following condition: with inoperable WT; with a solitary kidney; with synchronous bilateral WT; tumor thrombus in the inferior vena cava extending above the level of the hepatic veins; tumor involving contiguous structures whereby removing the kidney tumor requiring removal of the other organs, such as spleen, pancreas, or colon and with extensive pulmonary metastases. Preoperative chemotherapy by the COG has four regimes (Table 6). The agents for chemotherapy commonly are doxorubicin plus dactinomycin and vincristine; if with anaplastic histology, chemotherapy then includes regimen I (Table 6) [34].

**Table 6:** The Children's Oncology Group standard chemotherapy regimens for Wilms tumour.

REGIMEN NAME	REGIMEN DESCRIPTION	KEY FEATURES
**REGIMEN EE-4A**	Vincristine, dactinomycin × 18 weeks Postnephrectomy	Stage I/II FH WT
		Stage I focal or diffuse anaplasia WT
**REGIMEN DD-4A**	Vincristine, dactinomycin, doxorubicin × 24 weeks; baseline nephrectomy or biopsy with subsequent nephrectomy	Stage III/IV FH WT Stage II – IV Focal anaplasia
**REGIMEN I**	Vincristine, doxorubicin, cyclophosphamide, etoposide × 24< weeks postnephrectomy	Stage II – IV diffuse anaplasia
**REGIMEN M**	Vincristine, dactinomycin, doxorubicin, cyclophosphamide, and etoposide with subsequent radiation therapy<	–

The COG group has investigated five protocols. In COG 1 (1969–73) the vincristine + dactinomycin combination was more effective than either drug alone in stage II and III patients. COG 2 was conducted between 1974 and 1978. It found that a treatment duration of either 6 or 15 months was equally effective in stage I patients, and, after these results were published, treatment duration in protocols was shortened. Addition of adriamycin to the chemotherapy protocols improved the survival rate. In COG 3, stage I patients were treated successfully with a two-drug regimen for 10 weeks. For stage II patients, there was no significant difference in outcome between the RT or no RT arms, nor between the arm without adriamycin. Stage IV patients received no benefit from the addition of cyclophosphamide to the three-drug regimen. Different radiotherapy doses (1000 vs. 2000 cGy) also had no effect on survival. COG 4 also demonstrated that pulse-intensive actinomycin-D (single injection of 45 g/kg) was as potent as the long-term injection dose (15 g/kg/day for 5 days). The addition of adriamycin had a strong effect on survival in patients with stage III in COG 3–4 studies. COG 5 investigated whether stage I patients actually benefited from chemotherapy. Without chemotherapy, the 2-year overall survival was 100%, but relapse-free survival was 86% [35].

The COG recommends postoperative chemotherapy routinely used in all patients with WT except those at a very low risk: younger than 2 years at diagnosis with stage I favourable histology tumor weighing <550 g was sampled and confirmed negative lymph nodes.

**SIOP Studies:** The SIOP guideline recommends preoperative chemotherapy for all patients after diagnosis. For patients with unilateral localized tumor, 4-week pretreatment with vincristine (weekly) and dactinomycin (biweekly) is given; for patients with bilateral tumors, vincristine– dactinomycin for no longer than 9–12 weeks is recommended (doxorubicin is added for reinforcement in some patients); for patients with metastasis, a regimen including 6 weeks of vincristine–dactinomycin (like above) and doxorubicine on weeks 1 and 5 is given [34].

The SIOP 1 study compared the effectiveness of pre-nephrectomy irradiation versus immediate surgery and found that the two arms had the same overall survival rates. The SIOP 2 study found that preoperative treatment resulted in a decreased tumor rupture rate. In the SIOP 5 study, preoperative chemotherapy was substituted for preoperative radiotherapy. The SIOP 6 study showed that 17 weeks of chemotherapy treatment was as effective as 38 weeks of treatment for patients with stage I disease. Relapse risk increased in stage II lymph-node-negative patients who did not receive radiotherapy. The addition of epirubicin was planned in this group of patients. Also, radiotherapy doses were decreased from 30 to 15 Gy. The aim of the SIOP 9 protocol was to determine how the duration of preoperative chemotherapy affected survival. There was no significant difference in survival between 4 and 8 weeks of preoperative treatment. SIOP 93-01 studies have aimed to reduce treatment duration. Stage I patients were treated postoperatively for 4 weeks, whereas patients in other stages received 27 weeks of postoperative treatment. In this randomized study, there was no significant difference in terms of event-free survival rates, although patients with progressive disease during preoperative chemotherapy had poorer survival than the others [36]. The SIOP recommends postoperative chemotherapy in all patients with WT except those with stage I lowrisk tumor.

**UKCCSG Protocols:** This group treated patients with the postoperative chemotherapy regimen used by the COG group. This group used to perform biopsy prior to chemotherapy in all patients to ensure WT pathology, but because the rate of finding something when chemotherapy would be changed was <5%, this practice has been abandoned. Now this group follow SIOP exclusively. Patients with unresectable tumors were given preoperative chemotherapy. In patients with stage I, vincristine alone was as effective as vincristine and actinomycin-D. In the first study, the duration of the vincristine regimen in stage I was 6 months. This duration was shortened to 10 weeks in the second study [37]. This recommendation was limited to patients younger than 4 years. The group did not recommend using single-agent vincristine in older patients. Treatment results with stage IV patients were not as good as those obtained by the COG group.

Newborns and all infants less than 12 months of age require a reduction in chemotherapy doses to 50% of those given to older children. This reduction diminishes toxic effects reported in children in this age group while maintaining an excellent overall outcome. Liver function tests in children with WT should be monitored closely during the early course of therapy based on hepatic toxic effects (veno-occlusive disease) reported in these patients. Dactinomycin should not be administered during radiotherapy. Children treated for WT are at increased risk for developing second malignant neoplasms. This risk depends on the intensity of their therapy, including the use of radiation and doxorubicin, and on possible genetic factors. Congestive heart failure has been shown to be a risk in children treated with doxorubicin. Efforts, therefore, have been aimed toward reducing the intensity of therapy where possible [31]. Under the current COG study, children with stage III-V diffuse anaplasia are treated with a new chemotherapeutic regimen combining vincristine, doxorubicin, cyclophosphamide, and etoposide (Regime I) in an attempt to further improve the survival of these high-risk groups. All these patients receive radiation therapy to the tumor bed.

(d) Radiotherapy

Wilms tumor is a highly radiosensitive tumor. The postoperative radiotherapy (RT) is started within 10 days of surgery because delay beyond 10 days leads to tumor cell repopulating and chances of relapse increase. It has been shown that appropriate adjuvant RT reduces the postoperative recurrence to 0–4% in children with favourable histology. The dose of radiotherapy has decreased to approximately 10 Gy from the doses of 25–30 Gy that were recommended in the past. In the early years, all stage I and II patients were treated with flank irradiation, and those with stage III and IV were treated with whole abdominal radiotherapy. Since 1975, patients with favourable histology stage I no longer receive radiotherapy. Stage III and IV patients and those with otherwise local stage I and II receive flank irradiation instead of whole abdomen radiotherapy. Dosages were reduced to 2700 cGy and later to 1000 cGy depending on the histology and stage, rather than the age of the patient. Whole lung irradiation of 12 Gy was generally given in patients with metastatic lung disease with post-stamp boost (or boosts) of 10 Gy whenever possible [27]. Since 1990, patients with stage III and IV are treated with radiotherapy delivered to the tumor bed in 10-Gy dosages. Lung irradiation is used only in patients with residual or resistant disease after undergoing induction chemotherapy. The radiotherapy dose has varied from 10Gy to 40Gy. However, the use of radiation has now been reduced due to the awareness and documentation of radiation related late effects (growth disturbances, second cancer) in growing children of WT. The COG group has redefined the role of radiotherapy (Table 7) and has provided specific recommendations so that the minimum possible RT dose is administered. The COG-3 has documented that there is no survival difference at doses of 10Gy or 20Gy in stage III, FH group. The recommended dose per fraction is 1.2–1.5 Gy and it should not exceed 1.8Gy per fraction with concomitant chemotherapy [38].

**Table 7:** Guidelines for Radiotherapy in Wilms tumour.

CURRENT INDICATIONS OF RADIOTHERAPY	
•Stage II, III, IV with unfavourable histology	
•Stage III & IV with favourable histology	
•Metastatic disease	
**COG GUIDELINES**	**SIOP GUIDELINES**
•The COG recommends postoperative radiation used to the tumour bed for all patients with tumour stage III.	•The SIOP recommends whole-abdominal radiotherapy for patients with intermediate-risk or high-risk histology tumors with major preoperative or intraoperative tumour rupture/Spill, or macroscopic peritoneal deposits but only flank radiotherapy in other stage III criteria.
	•Pulmonary radiotherapy is indicated for lung metastases lacking complete response until postoperative week 10.
	•Patients with a complete response after induction chemotherapy with or without surgery do not need pulmonary radiotherapy.
	•Patients with viable metastases at surgery or high-risk histology require pulmonary radiotherapy.
	•Whole-lung irradiation is recommended for patients who did not receive lung irradiation during the first-line treatment, irrespective of histology.

The COG recommends postoperative radiation used to the tumor bed for all patients with tumor stage III. The SIOP recommends whole-abdominal radiotherapy for patients with intermediate-risk or high-risk histology tumors with major preoperative or intraoperative tumor rupture, or macroscopic peritoneal deposits; pulmonary radiotherapy is indicated for lung metastases lacking complete response until postoperative week 10. Patients with a complete response after induction chemotherapy with or without surgery do not need pulmonary radiotherapy. Patients with viable metastases at surgery or high-risk histology require pulmonary radiotherapy. Whole-lung irradiation is recommended for patients who did not receive lung irradiation during the first-line treatment, irrespective of histology.

(e) Treatment of Inoperable tumors

Since imaging studies alone carry the risk of overstaging, COG recommends determining ‘inoperability’ at surgical exploration. Tumors with caval extension above the hepatic veins or so massive in size that are considered risky to remove surgically should be treated with preoperative chemotherapy. Additionally, if tumors do not shrink after initial chemotherapy, open biopsy is indicated. Radiation is not used prior to surgery ever in WT treatment. If surgery is performed in a patient with caval or atrial extension, care should be taken to ensure that appropriate resources are available for paediatric cardiopulmonary bypass. In rare cases, advanced right-sided tumors may extend into the liver and wedge resection en bloc or even hepatic lobectomy may be necessary in these patients. If the diaphragm has been infiltrated by tumor, it should also be partially excised en bloc. Patients considered to have unresectable tumor based on imaging studies only should be considered stage III and treated accordingly. On the COG-5, these patients are treated after biopsy by initial chemotherapy with vincristine and dactinomycin with or without doxorubicin. If no reduction in tumor size has occurred after using 3 drugs, surgery is performed as soon as sufficient tumor shrinkage has occurred, generally within 6 weeks of diagnosis. Patients are subsequently treated as for stage III tumors, which includes postoperative radiation therapy. Because of the 5%–10% error rate in preoperative diagnosis of renal masses after radiographic assessment, confirmation of the diagnosis by open biopsy should be obtained prior to chemotherapy [31].

(f) Treatment of Anaplastic Wilms Tumor

All patients except stage I should be treated with intensive chemotherapy and radiotherapy. Vincristine + actinomycin-D + adriamycin and cyclophosphamide are used in this type of tumor. In the last COG study, patients with stage I disease were treated with vincristine + actinomycin-D for 18 weeks and achieved good results. Patients with diffuse anaplastic stage II–IV disease were treated with vincristine + cyclophosphamide + actinomycin-D + etoposide for 24 weeks. The results in this group were unsatisfactory. New drugs, such as carboplatin, should be tried in patients with anaplastic WT [39].

The risk factors associated with relapse are unfavourable histology, lymph node involvement, and age more than 6 years, diffuse spill, capsular and vascular invasion, and aneuploidy. The 2-year survival rate for children after local recurrence is 43%. The combination of ifosfamide, etoposide and carboplatin has demonstrated efficacy in this group of patients, but significant hematologic toxic effects have been observed. While very high-dose chemotherapy followed by autologous bone marrow transplant has been utilized in the past, a recent POG/CCG intergroup study used a salvage induction regimen of cyclophosphamide and etoposide (CE) alternating with carboplatin and etoposide (PE) followed by delayed surgery. Disease-free patients were assigned to maintenance chemotherapy with 5 cycles of alternating CE and PE, and the remainder of patients to ablative therapy and autologous marrow transplant. All patients received local radiation therapy. The 3-year survival was 52% for all eligible patients, while the 3-year survival was 64% and 42% for the chemotherapy consolidation and autologous marrow transplant subgroups, respectively. Patients in whom such salvage attempts fail should be offered treatment on available phase I or phase II studies [31].

(g) Treatment of Bilateral Wilms Tumor

**Fig. 7:**
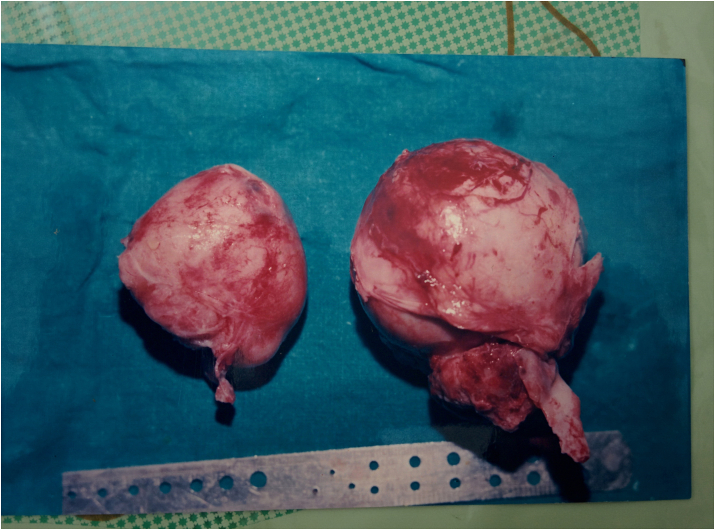
Photograph showing bilateral radical nephrectomy specimens of a patient with bilateral Wilms tumor with no normal residual kidney tissue on any side.

Fascinatingly, 5–10% of patients present with bilateral Wilms tumor (BWT), which may present as synchronous or metachronous bilateral tumors [1,2]. In the COG studies, approximately 4–6% of children registered, presented with synchronous bilateral tumors (Fig. 2, Fig. 7). The male-to-female ratio was 1:2, and the patients were usually younger at diagnosis. It was found that more bilateral or multifocal tumors occur at an earlier age (2 years versus 3.6 years in sporadic tumors). Also, the frequency of genitourinary anomalies (16%) and hemihypertrophy (5.4%) was higher compared to unilateral disease.

Historically, the management of bilateral Wilms tumor (BWT) was non-standardized and suffered from instances of prolonged chemotherapy and inconsistent surgical management which resulted in suboptimal renal and oncologic outcomes. Because of the risk of end-stage renal disease associated with the management of BWT, neoadjuvant chemotherapy and nephron-sparing surgery have been adopted as the guiding management principles. This management strategy balances acceptable oncologic outcomes against the risk of end-stage renal disease.

The presence of synchronous bilateral disease requires alteration of management. It is not recommended to perform unilateral nephrectomy and contralateral heminephrectomy as was the approach earlier. Under Children's Oncology Group (COG) protocols, unilateral WT is typically treated by up-front radical nephroureterectomy, with acceptable rates of long-term end-stage renal disease (<1%). Application of this strategy to patients with bilateral disease would render them anephric, and thus nephron-sparing approaches were developed and refined to preserve kidney function. Surgical strategy therefore attempts to preserve renal mass to minimize the risk of late renal failure. A recent multi-institutional Children's Oncology Group study (AREN0534) has confirmed the benefits of standardized 3-drug neoadjuvant chemotherapy and the utilization of nephron-sparing surgery in BWT patients; however, less than 50% of patients underwent bilateral nephron-sparing surgery.

The diagnosis of BWT is typically confirmed by the presence of bilateral renal masses, in an appropriately aged child, on ultrasound followed by contrast-enhanced CT abdomen/pelvis. A CT chest should also be performed to evaluate for pulmonary metastases at diagnosis, the most common site of metastatic disease in children with WT. Because other pediatric renal tumors are almost never bilateral, neoadjuvant chemotherapy for presumed BWT should be initiated without first performing a biopsy of any of the tumors [11]. In the recently completed first multicenter study specifically for BWT patients, the COG AREN0534 trial, intensification of neoadjuvant chemotherapy to three-drugs with vincristine, actinomycin-D, and doxorubicin (VAD) resulted in improved 3-year event free and overall survival compared to historical BWT patients treated on the NWTS-5 protocol who were often treated with only vincristine and actinomycin-D [11,30]. The rationale for up-front intensification of therapy was to achieve improved tumor response to facilitate bilateral nephron-sparing surgery. The feasibility of bilateral nephron-sparing surgery should be assessed after six weeks of VAD therapy by contrast-enhanced CT scan of the abdomen/pelvis. If more than 50% volume reduction has been achieved for all tumors, but nephron sparing surgery is still not feasible, neoadjuvant VAD should be continued for six more weeks. Surgical resection should be performed regardless of tumor status at the 12-week timeframe. If less than 50% volume reduction has been achieved after the six initial weeks of therapy for any tumor, and bilateral nephron-sparing surgery is still not feasible, open surgical biopsy of all tumors should be performed to assess for the possibility of alternative histologies such as diffuse anaplasia, blastemal predominance after neoadjuvant therapy, differentiated tumor without remaining viable/proliferative elements, or an alternate diagnosis (exceedingly uncommon). Core biopsy often misses the presence of diffuse anaplasia, which could be responsible for treatment resistance, and should thus be avoided in this treatment algorithm [40].

Approximately 10% of patients with bilateral tumors have unfavourable (anaplastic) histology and may benefit from more aggressive chemotherapy (addition of doxorubicin and cyclophosphamide) and radiation therapy and an aggressive surgical approach at the second-look operation. Salvage chemotherapy regimens using Cisplatinum, ifosfamide and VP-16 have been found to be helpful. After chemotherapy, the patient is reassessed with abdominal CT to determine the feasibility of resection. If serial imaging studies show no further reduction in tumor, a second look surgical procedure should be performed. For small synchronous bilateral lesions at the poles, bilateral partial nephrectomies or wedge resections can be performed. Excisional biopsy or partial nephrectomy is regarded as appropriate only if radical tumor resection is not compromised, negative margins are obtained and if two thirds of the renal parenchyma can be preserved The goal is to achieve survival and at the same time to preserve an adequate amount of renal parenchyma. In case of a large tumor on one side and a contralateral small one, radical nephrectomy on the extensively involved site and partial nephrectomy on the opposite side is done [40].

If conditions are not favourable for any surgical intervention, another biopsy is taken to confirm viable tumor. Chemotherapy and/or radiation therapy following the second-look operation is dependent on the response to initial therapy, with more aggressive therapy required for patients with inadequate response to initial therapy observed at the second procedure. A third look may be indicated; bilateral nephrectomy and subsequent renal transplantation remains the last option. Unfortunately, due to immunosuppression, recurrence of disease occurs frequently. Before considering bilateral nephrectomy, bench surgery with autotransplantation and intraoperative radiotherapy may be performed. The cumulative survival rate for infants with bilateral tumors is approximately 65–70% at 10 years. However, one series reported overall survival of metachronous bilateral Wilms tumor to be 49.1% and 47.2% at 5 and 10 years, respectively [16,31].

Metachronous bilateral tumors were reported in about 1.5% of COG patients. Since many of these lesions appear to be overlooked at initial laparotomy, a thorough investigation of the opposite kidney remains crucial. Children younger than 12 months diagnosed with Wilms tumors, who also have multicentric disease or NRs, in particular perilobar NRs, have a markedly increased risk of developing contralateral disease and require frequent and regular imaging of the contralateral kidney for several years. The median interval of diagnosis of metachronous WT ranges from 1.37 (COG) to 3.29 (SIOP) years. Though radiotherapy has been recommended in bilateral WT in reduced doses, the authors advocate avoiding radiotherapy in Bilateral WT and preferring salvaging chemotherapy schedules to prevent radiation nephritis and glomerulosclerosis. It has been seen by a long term evaluation of renal function in patients with irradiated bilateral WT that 34.6% have deranged renal functions with elevated urea and creatinine levels [41].

The principal treatment of bilateral WT is nephrectomy of the larger tumor after preoperative treatment or through immediate surgery [42]. After induction chemotherapy, the smaller tumor should be removed by partial nephrectomy. Limited radiotherapy could be applied. Whatever the treatment, salvage of the kidney should be the goal. Both the COG and SIOP recommend preoperative chemotherapy and resection for bilateral WT. Bilateral renal-sparing surgery can be done in patients with synchronous bilateral WT. Renal parenchyma sparing may help preserve the renal function in these children. Renal transplantation is recommended and is usually delayed until 1–2 years without evidence of relapse. The SIOP also suggests that preoperative chemotherapy should be limited to not longer than 12 weeks, with time intervals for evaluation fixed to 6 weeks [40,42].

Both the COG and SIOP recommends preoperative chemotherapy and resection for bilateral WT. Bilateral renal-sparing surgery can be done in patients with synchronous bilateral WT. Renal parenchyma sparing may help preserve the renal function in these children. Renal transplantation is recommended and is usually delayed until 1–2 years without evidence of relapse. The SIOP also suggests that preoperative chemotherapy should be limited to not longer than 12 weeks, with time intervals for evaluation fixed to 6 weeks [34]. A recently completed multi-institutional COG study (AREN0534) has standardized the approach to neoadjuvant therapy and timing of surgical resection, resulting in great benefit to patients with BWT.

### 3.9 Renal transplantation for Bilateral Wilms Tumor

Bilateral nephrectomy may be required in BWT patients because of disease relapse or complications of surgical or medical therapy, necessitating hemodialysis and eventual renal transplantation. Also, bilateral nephrectomy will likely eventually be required for WT patients with Denys–Drash syndrome, who eventually develop nephropathy and end-stage renal disease. Transplantation has historically been delayed until 1–2 years after cancer therapy because most WT relapses occur within 2 years of diagnosis. However, more recent data show WT patients, even those who underwent early transplant, have outcomes similar to other renal transplant patients. Given the morbidity and mortality associated with chronic pediatric dialysis, consideration of earlier transplantation should be made in WT patients with end stage renal disease and no evidence of cancer.

The current treatment strategy for BWT balances acceptable oncologic outcomes with the goal of maintaining the maximum amount of functioning renal parenchyma. It is imperative that the pediatric surgeon be intimately aware of the treatment algorithm for BWT when managing such patients. This algorithm is characterized by avoidance of initial tumor biopsy, three-drug neoadjuvant chemotherapy (VAD), assessment of tumor volumetric response at 6 weeks, a decision whether to continue VAD, perform an open biopsy, or change chemotherapy for a subsequent six weeks, and then to perform surgical resection in all cases at 12 weeks of therapy. Close collaboration between pediatric surgeons and the multidisciplinary oncology team is necessary to negotiate this complex algorithm and to maximize the chances that BWT patients have optimal long-term oncologic and renal outcomes [40].

(h) Treatment of Recurrent WT

The recurrence rate in patients with familial hypercholesterolemia WT is about 15% and patients with anaplastic histology is about 50%. The leading locations of relapse are the lung, abdomen/flank and liver. The prognosis and selection of further treatment for patients with recurrent WT depend on many factors, including the site of recurrence, tumor histology, length of initial remission, and initial chemotherapy regimen (2 versus 3 drugs). Historically, the mortality rate of patients with recurrent favourable histology, WT ranges from 25% to 40%. Outcome has recently improved to 60% in patients with relapse [43]. The COG guideline has categorized the patients with recurrent WT into three risk groups: standard risk, high risk and very high risk. For standard-risk relapsed WTs, surgery is given when feasible; radiation therapy and chemotherapy (alternating courses of vincristine/doxorubicin/cyclophosphamide and etoposide/cyclophosphamide) are given. For patients with high risk and very high risk relapsed WTs, chemotherapy (alternating courses of cyclophosphamide/etoposide and carboplatin/etoposide), surgery, and/or radiation therapy and hematopoietic stem cell transplantation are recommended. The SIOP classifies the patients with recurrent WT into group AA, group BB and group CC. For patients in group AA, only vincristine and/or dactinomycin (no radiotherapy) is adopted as the first-line treatment, containing four drugs in the regime (combinations of doxorubicin and/or cyclophosphamide and carboplatin and/or etoposide); for group BB, an intensive reinduction regimen is given (including the combination of etoposide and carboplatin with either ifosfamide or cyclophosphamide), followed by either high-dose melphalan and autologous stem cell rescue or two further reinduction courses; for group CC, camptothecins (irinotecan or topotecan) or novel compounds are recommended [13,26].

(i) Lung metastases

Pulmonary nodules seen on chest CT and not on chest radiograph (‘CT only’ metastases) do not mandate treatment with whole-lung irradiation in COG-5. COG-4 data raise the possibility that children with CT-only pulmonary nodules who receive whole lung irradiation have fewer pulmonary relapses than those who were treated less aggressively (based on the extent of locoregional disease with 2 or 3 drugs), but a greater number of deaths due to treatment toxicity (4-year event-free 89 vs. 80%, overall survival 91 vs. 85%). Lung nodules should be treated with lung irradiation. There are some data on rapid early responders who clear their lungs with chemotherapy alone and lung irradiation may be omitted, but in general, persistent lung lesions require Irradiation. The nodules should be removed to confirm diagnosis [31].

(j) Infants with WT

The most common solid renal mass in infants <6 months old is Congenital Mesonephric Nephroma (CMN). Additionally, WT in this group does not necessarily need adjuvant chemotherapy should they meet very low risk requirements above. The SIOP recommends primary nephrectomy for infants younger than 6 months (182 days) unless tumors are judged not suitable to immediate nephrectomy. Postoperative chemotherapy is similar for infants to that in older patients undergoing direct nephrectomy, with drug doses adjusting according to age and body weight [44].

Differential Diagnosis of Wilms Tumor.

(a) Clear Cell Sarcoma of the Kidney (CSSK)

CCSK accounts for approximately 3% of renal tumors. Its location, clinical presentation, gross appearance and age at diagnosis are same as of WT. It is also called as an unfavourable histologic variant of WT with poor prognosis and was called “bone metastasizing renal tumor”. The incidence peaks during the second year of life (COG Mean age at presentation: 36 months, Range: 2 months - 14 years). The male to female ratio is 2:1. It has distinctive histopathologic features, a much higher rate of relapse and death than in favourable histology WT. The histopathologic characteristics include a wide diversity of features, ranging from spindle cell to epitheloid patterns. Most tumors show the classic histological picture, i.e. multiple blended patterns. The following histopathologic variants were described: myxoid, sclerosing, cellular, epithelioid, palisading, spindle cell, storiform, and anaplastic pattern.

Bone metastasis is the most common mode of relapse, followed by lung metastases, local (abdominal/retroperitoneal) recurrence, and brain metastases. CCSK metastases are frequently encountered in unusual soft tissue (e.g. scalp, epidural, nasopharynx) and other sites (orbital). The time interval to relapse ranged from <16 months to 4 years. Although the overall relapse rate is significantly lower for patients treated with doxorubicin, the risk of recurrence is prolonged.

Currently (COG-5), patients with CCSK are treated with initial nephrectomy regardless of stage, abdominal radiation (10.8 Gy) and combined chemotherapy with actinomycin D, vincristine and doxorubicin. The main prognostic factors for favourable outcome in CCSK are revised stage 1, age at diagnosis (2–4 years), therapy with doxorubicin and absence of tumor necrosis. Moreover, Stages I-III patients do very well [45].

(b) Rhabdoid tumor of kidney (RTK)

It was initially regarded as a solid monophasic, or rhabdomyosarcomatoid variant of unfavourable histology WT. It is now recognized as a separate highly malignant entity. RTK represents only 1.8% of cases entered into COG since 1969, with a median age at presentation of 17 months and a slight male preponderance (male-to-female, 1.5:1). In about 15% of RTK, patients develop other primary embryonal tumors in the midline posterior fossa, particularly medulloblastoma. These intracranial tumors are histologically distinct from the primary renal lesion. In contrast to WT, about 80% of RTKs have stage III or IV disease at presentation. Grossly, they are bulky, solid and relatively well-circumscribed lesions. The histiogenesis remains controversial. Deletion of the hSNF5/INI1 gene on chromosome 22 has been found in all of these tumors. The tumor behavior is extremely aggressive and clinical management (triple chemotherapy) has not proven successful. So far, male sex and high tumor stage are the only identified unfavourable prognostic indicators. Metastases occur most frequently in the lung (70%) and Brain and most patients with relapse die from tumor progression (COG 96%). That is why Brain Imaging is required at the time of Diagnosis. The reported survival rate at 3 years is less than 20% [46].

(c) Congenital Mesoblastic Nephroma (CMN)

About 2.8% of all renal neoplasms in children are CMNs. It is the most common benign renal tumor in neonates and a low grade spindle cell tumor which arises from the renal medulla. It is also known by other names like fetal renal hamartoma, leiomyomatous hamartoma and mesenchymal hamartoma of infancy. This tumor is the most common in infants <6 months and that Lymph Node sampling is mandatory for staging and in case of non-CMN pathology. With the increasing use of antenatal USG, many cases of CMN have been detected in utero. There is an increased association with prematurity and polyhydramnios. Nearly all solid renal tumors presenting in the first week of life are mesoblastic nephromas. However, a few cases have been reported in older children. Mean age of diagnosis of 3.4 months with a male preponderance (male-to-female ratio 1.8:1). Hypertension, increased renin concentration and skeletal fibromatosis have been reported. On USG, it presents as an evenly echogenic mass with concentric echogenic and hypoechoic rings resembling uterine fibroids. Haemorrhage and cyst formation secondary to central regions of necrosis may occur with time. Calcification is rare. Grossly, it is a light tan, fleshy with a whorled configuration and has ill-defined peripheral borders, blending into the adjacent renal parenchyma and even the perirenal fat. Most are centred near the hilum of the kidney. Microscopically, it consists of monomorphic spindle-shaped cells, resembling fibroblasts with scant interstitial collagen. Two morphological subtypes are distinguished: the classical or leiomyomatous type and the atypical or cellular type. Mixed forms have also been described.

Despite excellent prognosis, local recurrence and even tumor-related deaths have been described and were always related to the cellular (atypical) form or to the mixed form, particularly in patients aged more than 3 months and in those cases where surgical removal was not complete. Cytogenetics have reported common trisomies in cellular CMN, particularly of chromosome 11 and t (12; 15) (p13; q25)-associated ETV6-NTRK3 gene fusions. Total surgical excision independent of histological type without further therapy is recommended for most patients as the treatment of choice. Tumor rupture and difficulties in achieving clear surgical margins have been frequently reported but did not affect the excellent prognosis [47].

(d) Intrarenal neuroblastoma and intrarenal teratoma

Neuroblastoma affects mainly aged between 2 months and 2 years and are slightly more common in Caucasian boys. This tumor in most cases resolves spontaneously, leaving just a focus of fibrosis or calcification in adults. Intrarenal neuroblastomas are rare tumors and pose diagnostic challenges. Clinically and radiologically, they are indistinguishable from WT. Elevated urinary vanillylmandelic acid (VMA) levels and serum NSE should allow a differential diagnosis before surgery. The prognosis is grave. While sacrococcygeal teratomas contain elements of WT and WT have been found to produce Alpha-fetoprotein. Few cases of intrarenal teratomas have also been described. The diagnosis depends on histological examination. Teratoid WT is an unusual variant of nephroblastoma, in which there are different types of cells and tissues along with areas of WT [46]. After complete resection, the prognosis should be excellent provided the tumor does not contain yolk sac elements.

Prognosis.

The prognosis of WT is the most favourable among all solid tumors. The survival rate is 95% in patients in stages I and II, 75–80% in stage III patients and 65–75% in patients with stage IV. WT can be classified into favourable and anaplastic histology groups for prognostic purposes. Only 15% of patients with favourable histology have recurrent disease, compared to 50% in those with anaplastic histology. The most common sites of recurrence are the lungs, pleura, tumor bed and the liver. Among all patients with WT, those with liver involvement have a poor prognosis as compared to those with lung metastasis. Diffuse anaplasia confers poor prognosis, has chemotherapy resistance and may still be present after preoperative chemotherapy, however; children with stage I anaplastic tumors (Stage I Anaplasia) have an excellent prognosis. Stage V patients have a 4-year survival rate of 94% for those with the most advanced lesion of stage I or stage II, and 76% for those with the most advanced lesion of stage III [48,49]. Thus, the important prognostic factors for WT are:

1. Stage of the disease
2. Favourable or unfavourable histology
3. Metastases at presentation
4. Regional lymph node involvement
5. Hyperdiploidy which correlates well with anaplastic variety

### 3.10 Long term complications of WT

Fortunately Wilms tumor is a curable malignancy, but iatrogenic sequelae are possible. Paulino et al. reported late effects of therapy in more than two thirds of children treated for WT [50]. Besides morbidity from chemotherapeutic agents, potential side effects of radiotherapy like intestinal strictures, ulceration, perforation, haematochezia, growth arrest and osteonecrosis have to be considered [50].

(a) Renal function

COG and SIOP studies showed that the risk of renal failure for patients with unilateral WT and a normal opposite kidney is very low (0.25%). Most of these children had unrecognized renal disease (Denys-Drash syndrome) followed by radiation nephritis. In patients with nephrectomy and abdominal irradiation, renal dysfunction is more common. However, the development of compensatory post-nephrectomy hypertrophy of the contralateral kidney is obvious and proteinuria and hypertension may occur long after nephrectomy. ‘Renal failure’ in these patients is most often caused by bilateral nephrectomy followed by radiation nephritis and surgical complications. The DTPA clearance after unilateral nephrectomy for WT was found to be normal. However, microalbuminuria in 24-h urinary collections has been detected in 84% of the patients, indicating evidence of hyperfiltration injury [51]. This highlights the need for close monitoring of the renal function of long-term follow-up patients after WT in addition to the routine monitoring for tumor recurrence.

(b) Lung damage

Both chemotherapeutic agents and total lung irradiation can cause severe changes in pulmonary function. Prophylaxis against Pneumocystis carinii is recommended for patients receiving pulmonary irradiation.

(c) Congestive heart failure

Congestive heart failure is typically seen after administration of anthracyclines. Reported cardiotoxicity includes electrocardiographic changes, changes in myocyte morphology (necrosis and fibrosis), decreased cardiac function and congestive heart failure. Dose related cardiomyopathy caused by doxorubicin is a well-known complication, reported for approximately 5% of patients receiving a cumulative dose of 400–500 mg/m^2^. MUGA scans can be used to assess left ventricular ejection fraction (LVEF) and myocardial movements and thus timely discontinuation of doxorubicin can prevent congestive heart failure [51].

(d) Liver damage

COG-4 studies reported a dose-related incidence of hepatotoxicity in patients receiving chemotherapy (especially vincristine and actinomycin D). Irradiation also increases the risk for hepatotoxicity and veno-occlusive disease as characterized by hepatomegaly, elevated liver enzymes, hyperbilirubinemia and ascitis [51].

(e) Infertility

Damage to the reproductive systems may occur as late sequelae of both, gonadal radiation or chemotherapeutic agents. Radiation effect even on prepubertal germ cells may lead to hormonal dysfunction (hypogonadism) or infertility [51]. Vincristine is a major risk factor for azoospermia.

(f) Second malignant neoplasms

The risk of developing a second malignant neoplasm in patients with successfully treated WT is 1.6–5.6% [51]. Tumors mainly seen in the irradiated field are hepatocellular, bone, breast and thyroid malignancies.

(g) Musculoskeletal function

Scoliosis & musculoskeletal abnormalities have been found more frequently in irradiated patients than in those patients who did not receive radiotherapy including lower rib hypoplasia and limb length inequality. Abdominal radiation can also produce significant reduction in sitting height and a more modest decrease in standing height. These effects are more pronounced the younger the patient is at the time of radiotherapy. Flank and abdominal radiotherapy doses of 20–30 Gy produce a height loss calculated by age at treatment. For a child aged 1 year this was 9 cm, aged 5 years 7 cm and aged 10 years 5.5 cm [51]. Ionizing radiation has well been documented to interfere with epiphyseal growth.

Follow up.

After completion of therapy, the frequency of imaging is dependent on the stage and histology of the tumor. Moreover, physical and laboratory tests coincide with the schedule for imaging. In general, all patients are reviewed every 3 months for the first year, and then every 6 months for another 2 years. During each of the follow-ups in the first three years it is recommended to get a radiological evaluation. This may be an ultrasound or CECT scan in addition to a chest x-ray [25]. The likelihood of recurrence after the first three years is less; however these patients should be followed up every year for various long-term complications.

Major challenges in Developing Countries.

Challenges faced by developing countries include huge population with large number of cases, poverty, malnutrition and presentation of the disease in advanced stages (huge bulky tumor) coupled with noncompliance with schedule and limited facilities for advanced surgery and supportive services that are necessary for proper management of these cases.

### 3.11 Future perspectives

Recent advances in understanding the molecular biology of the tumorigenesis of WT have provided significant implications for the clinical management. Thus, both large study groups (COG & SIOP) currently aim to intensify treatment for patients with poor prognosticators while reducing therapy and subsequent long-term complications, for those with favourable prognostic features. Parenchymal sparing renal surgery for patients with small unilateral WT remains controversial. Treatment of children with Wilms tumor should certainly involve a team of specialized paediatric surgeons, oncologists, radiologists, pathologists and radiotherapists. Partial nephrectomy or nephron-sparing surgery should be done in selected patients. Low-risk patients should receive fewer chemotherapeutic agents and at lower cumulative doses. In COG these patients receive no adjuvant therapy. Trials to further reduce radiotherapy doses or omit radiotherapy in selected cases may be undertaken.

## 4 Conclusion

Most of the patients with WT have good prognosis owing to multimodality treatment and multidisciplinary care. But further studies should be done on usage of chemotherapy and radiotherapy under more accurate risk-stratified strategies and to decrease the late effects of surgery.

## Consent

The patients have given their consent for the study to be published.

## Figure permission

Permission has been obtained from the parents for the photographs of the child to be potentially published.

## Funding

There was no funding from any source.

## Declaration of competing interest

We declare that we do not have any conflict of interest.
